# Diseases of the Digestive Organs

**Published:** 1895-11-09

**Authors:** 


### DISEASES OF THE DIGESTIVE ORGANS.

**Appendicitis.**—It is gradually becoming recognised that local causes and injuries cannot explain all cases of this affection. Sutherland,[1] in discussing the work that has been done on this subject, refers to Bland Sutton's theory that the appendix is an abdominal tonsil, arguing both from the excess of lymphoid tissue it contains, and from the similarity of the inflammatory attacks to which it is subject. From it are produced numbers of leucocytes which destroy the microbes found in such hosts about that part of the intestinal tube. Frequently the primary change in the disease is an inflammation of the tissues of the appendix, and without constriction or ulceration.

This may be caused by a poison such as that of rheumatism, and the effect of the inflammation is that the protective power of the appendix is lessened, micro-organisms gain entrance, and a septic state follows, just as in rheumatic tonsilitis. Sutherland has previously adduced cases in which manifestations of gout or rheumatism alternate or coincide with appendicular symptoms. The lymphoid tissue in early life is very active, and it is then that we most often meet with attacks. Chills, too, are frequent precursors, over-eating, general aching pains, and malaise may precede others, even an attack of tonsilitis may be the first symptom before appendicitis appears. Full doses of salicylates at the outset will sometimes cause the abortion of the disease, and the author would even add two or three grains of calomel. Brazil[2] reported two cases of rheumatic appendicitis, in one of which articular symptoms were also present. Both recovered quickly under salicylates. J. E. Blomfield[3] mentioned a family which suffered severely from rheumatic attacks, and out of them two developed appendicitis; one of them was rapidly cured by salicylates. T. E. Frazer[4] speaks of having attended several others which yielded to the same treatment; but, of course, the diagnosis is open to question in many of these cases. Though some such poison as the rheumatic may cause the first stage of an attack, infection by streptococci or the colon bacillus soon follows, and exudation produces compression of the tissues. Now in the narrow tube of the appendix this may cause necrosis from tension, or at least render the anæmic tissues still more vulnerable to bacteria. R. J. Morris[5] believes that infection by the colon bacillus only produces a temperature of 100 deg. or 101 deg., while with streptococci a much higher degree is reached. Even mild cases may cause, among other complications, embolic abscesses in the liver, and this when appendicitis has not been suspected. At the German Congress of Internal Medicine a warm controversy took place between those who, like Sonnenberg, would differentiate between catarrhal and suppurating typhlitis, and the adherents of Sahli, on the other hand, who regard all forms of typhlitis as due to a purulent inflammatory focus in the appendix. The latter school insist on the infective origin of every case, and the inability of coprostasis to produce the disorder. Still, Sahli confesses that spontaneous recovery does often occur, and where signs of suppuration have not appeared, advises medical treatment at first. He uses the ice pack, small doses of opium, rectal feeding, and leeches, but watches without ceasing for the first symptom of suppuration to advise operation. Sonnenberg[6] agrees that in deferring operation there must be no great change present in temperature and pulse, or in facial expression. However, many fatal cases have shown little alteration in any of these respects, and Henrotin[7] warns us also to watch the tumour in the flank that it remains well defined, deeply situated, and not variable in size or consistence, otherwise to operate without delay.

The question of giving aperients in mild cases is an important one. W. Schell[8] and many others hold that neither salines nor cathartics are desirable even in the mildest cases, for the bowel can be unloaded of decomposing matter by an enema without equal risk of opening perforations and breaking down protective adhesions. Indeed, this seems to be now the generally accepted view, though some of those who believe in a simple catarrhal inflammation still employ aperients in the earlier stages. Less importance is attributed than formerly to Concretions. Rochaz[9] has, however, collected copious statistics as to their frequency, and thinks they have much to do in causing the affection. Some operators have found them in the majority of cases, and others in only 1 or 2 per cent.

**The Duodenum.**—Various forms of cancer may occur primarily here; the diagnosis is difficult, as the disease may simulate pyloric stenosis, or cancer of the lower bowel. In some cases there is permanently a return of the bile and pancreatic juice into the stomach.[10] Simple ulcer is generally found close to the pylorus, and is single, round, and specially apt to cause perforation if situated on the posterior wall of the bowel. The pain felt comes on usually from two to four hours after a meal. Letulle[11] holds that ulceration is often unsuspected, and found it in 8 per cent. of post-mortems. Perry and Shaw,[12] in the Guy's Hospital Reports, discovered simple ulcers in ·4 per cent. only, and these were three times more frequent in males than in females. In those dying of burns ulcers occur to the extent of 3·3 per cent., and, according to some authorities, are due to the absorption of pyridine produced by the burnt fat. Perry and Shaw regard them as septic in origin. Other septic states, such as gangrene and bed-sores, are often accompanied by similar ulcers. They found 16 cases in which ulcers accompanied Bright's disease.

**Colitis.**—Hale White[13] draws attention to three forms of this disease—simple, membranous, and ulcerative. The two former may be chronic. Milk diet, rest in bed, bismuth, and catechu are valuable in the simple form. In the membranous and ulcerative types all treatment is unsatisfactory; the connection with Bright's disease and gout, and the possible production of liver abscesses must be remembered. Several cases of membranous colitis have been detailed by C. P. Crouch,[14] who emphasises the uselessness of treatment, while noticing that the health may be little affected by the disease. Wilson[15] advises acid. hydrocyan. dil. m. iii. after food. F. C. Wood[16] found Dr. Ralfe's prescription of a drachm of castor oil at night and m. x. of ol. terebinth every morning efficacious.

[Many other cases have been recently reported, and the abstractor recently met with one following after recovery from typhoid which resisted every form of treatment.]

**Dysentery.**—W. C. Weber[17] recommends small doses of calomel, followed by bismuth and salol, injections of ice-cold water, and, for the chronic form, mentions injections of nitrate of silver or quinine. Gassar[18] regards the anguillula stercoralis as a frequent cause, and doubts the importance of the amœba coli, since the injection of sterile vegetable matter produced attacks similar to those where the amœba was found. De Silvestri[19] showed that a certain diplococcus could also cause it. In a minute discussion of the treatment of every type of dysentery, Sir J. Fayrer[20] emphasises the value of ipecacuanha, dieting, and, in chronic cases, of turpentine, bael fruit, and especially of a change of climate. A plain milk diet, with rest in bed, will sometimes effect a cure in intractable cases.[21] G. Thin[22] finds that, where this treatment is difficult because the patients cannot swallow enough milk, milk evaporated to half its bulk with frequent stirring can be often taken with success, and forms an invaluable food. H. Gallay[23] relies on a daily injection of nitrate of silver, gr. xx. to the litre, after washing out the bowel. This should be retained for two or three minutes, and used daily for two months. No special diet or medicine is required.

**Minor Ailments.**—Morning diarrhœa may possibly be a form of colitis. F. Delafield has noticed that it is far from uncommon and may go on for years. Some of the most obstinate cases pass much mucus in the stools. Castor oil in five or ten drop doses has seemed to him to do much good. W. H. Draper found that a nitrogenous diet without any sugar or fruit generally brought relief. On the other hand, Beverley Robinson believed the affection to be malarial, and recommended bark and arsenic.[24] J. C. Wise regarded it as the result of dyspepsia in the upper part of the digestive tract and recommends naphthol and change of air.[25] Summer diarrhœa appears closely connected with the temperature of the soil, for Priestly has noticed when it rose above 56 degrees the mortality from diarrhœa soon commenced.[26] Tannigen, as a remedy for chronic enteritis, has been tried by C. Künkler, who found it very valuable in daily doses of three to seven grains in boiled water.[27] Rectal injections or lavage by two or three quarts of water containing twenty grains of sulpho-carbolate of zinc are also recommended by a writer in the *Therapeutic Gazette*, April 16th. The value of naphthol-bismuth in stopping fermentation and other changes in the intestines is strongly urged by H. Engel, who quotes the investigations of Von Fincke and others, and reports many cases showing the powerful antiseptic action of this drug on the intestines.[28] Minute repeated doses ($\frac{1}{50}$ to $\frac{1}{20}$ grain) of calomel are again advocated by W. B. Stewart. He insists on the use of a pure drug, and has it carefully triturated with sugar of milk.[29] A number of cases of poisoning through overdoses of drugs administered by the rectum have led to special legislation in Germany,[30] and indeed the danger is one which must never be forgotten, as more constant use is made of rectal feeding and medication. Some doubt has been thrown on the activity of magnesium-sulphate when administered hypodermically, but both Fincke and James Wood have found it successful as a purgative in 70 per cent. of their cases. Wood, however, failed when using the tabloids of this drug, and succeeded with an ordinary neutral solution of two or three grains.[31] Among remedies for tapeworm the pelletierine of Tanret (9½ grammes of the sulphate) is recommended to be taken fasting with a little tannin. The usual aperients should be given, but the drug is only suitable for adults.[32] Masius notices the occurrence of amaurosis after repeated doses of male fern. Eichorst had previously met with this result, which he thought might be prevented by giving castor oil at the same time.[33]

[1] Lancet, Aug. 24, 1895 [2] B.M.J., May 25. [3] B.M.J., Sep. 14 [4] B.M.J., June 15. [5] N.Y.Med.J., Feb. 27. [6] Med. Week, Ap. 19. [7] Chicago Clin. Rec., Ap. [8] N.Y.Med.J., Ap. 20. [9] Med. Chronic, March. [10] Am.J.M.Sc., Ap. [11] Med. Chron., Dec. [12] Med. Chron., Jan. [13] Lancet, March 2. [14] Bristol M.C.J., Ap. [15] Lancet, Jan. 5. [16] Lancet, Jan. 12. [17] Med. Rec., March 30. [18] B.M.J., May 25. [19] Lancet, Feb. 16. [20] Pract., Dec., 1894. [21] Lancet, July 6. [22] B.M.J., Feb 9, [23] Lancet, Jan. 19. [24] Med. Record, May 11. [25] Med. Rec., June 22. [26] B.M.J., May 11. [27] Lancet, Ap. 20. [28] N.Y.M.J., March 30. [29] Med. Rec., May 11. [30] Am.M.S. Bulletin, Ap. 1. [31] Ther. Gazette, Jan. 15. [32] B.M.J., Feb. 16. [33] Med. Week, July 5.

